# Clinical Anterior Segment and Ocular Surface Findings Across Renal Replacement Modalities: Associations with CKD-Related Factors, Mineral Metabolism and Activin A

**DOI:** 10.3390/life16081294

**Published:** 2026-08-06

**Authors:** Ioana-Madalina Bilha, Stefana Catalina Bilha, Nada Akad, Adrian Covic, Simona Hogaș, Mihai Marian Hogaș, Ioana Alina Halip, Calina Anda Sandu-Boz, Camelia Margareta Bogdanici, Irina Draga Caruntu

**Affiliations:** 1 Department of Morpho-Functional Sciences I-Histology, “Grigore T. Popa” University of Medicine and Pharmacy, 700115 Iasi, Romania; ioana-madalina.bilha@email.umfiasi.ro (I.-M.B.); irina.caruntu@umfiasi.ro (I.D.C.); 2 Department of Internal Medicine II-Endocrinology, “Grigore T. Popa” University of Medicine and Pharmacy, 700115 Iasi, Romania; 3 Department of Internal Medicine II-Nephrology, “Grigore T. Popa” University of Medicine and Pharmacy, 700115 Iasi, Romania; akad_nada@d.umfiasi.ro (N.A.); adrian.covic@umfiasi.ro (A.C.); simona.hogas@umfiasi.ro (S.H.); 4 Department of Morpho-Functional Sciences II, Discipline of Physiology, “Grigore T. Popa” University of Medicine and Pharmacy, 700115 Iasi, Romania; mihai.hogas@umfiasi.ro; 5 Department of Internal Medicine III-Dermatology-Venerology, “Grigore T. Popa” University of Medicine and Pharmacy, 700115 Iasi, Romania; ioana-alina.grajdeanu@umfiasi.ro; 6 Department of Surgery II-Ophthalmology, “Grigore T. Popa” University of Medicine and Pharmacy, 700115 Iasi, Romania; calina-anda.sandu@umfiasi.ro (C.A.S.-B.); camelia.bogdanici@umfiasi.ro (C.M.B.); 7 Romanian Medical Science Academy, 030171 Bucharest, Romania

**Keywords:** chronic kidney disease, hemodialysis, kidney transplantation, ocular surface, tear break-up time, Schirmer I test, cataract, Activin A, mineral metabolism

## Abstract

Background: Chronic kidney disease (CKD) is characterized by persistent inflammation, metabolic dysregulation, and mineral and bone disorder (CKD-MBD), all of which may affect ocular tissues. While retinal manifestations of CKD have been increasingly investigated, data regarding clinical anterior segment modifications and their relationship with systemic biochemical parameters remain limited. Methods: We performed a cross-sectional study including 130 participants: 53 non-CKD subjects, 29 patients undergoing maintenance hemodialysis (HD), and 48 kidney transplant recipients (KTRs). All participants underwent comprehensive ophthalmic examination, including best-corrected visual acuity (BCVA), intraocular pressure (IOP) measurement using Goldmann applanation tonometry, Schirmer I test, tear break-up time (TBUT) and assessment of cataract and blepharitis prevalence. Serum creatinine estimated glomerular filtration rate (eGFR), calcium, phosphate, parathyroid hormone (PTH), magnesium, 25-hydroxyvitamin D, and Activin A were recorded. Associations between ocular and systemic parameters were evaluated using correlation and regression analyses. Results: TBUT, Schirmer I and IOP did not differ significantly among groups (all *p* > 0.05). After adjustment for age, sex, BMI and diabetes mellitus (DM), HD patients showed worse visual acuity than the non-CKD reference group, reflected by higher logMAR BCVA values (adjusted B = +0.128, *p* = 0.024). In KTRs, higher corrected calcium was independently associated with reduced tear film parameters, correlating with both shorter TBUT (B = −0.903, *p* = 0.037) and lower Schirmer I values (B = −5.947, *p* = 0.008). Circulating Activin A increased progressively from controls to KTR to HD patients (median 189.5 vs. 273.0 vs. 314.0 pg/mL, *p* < 0.001) but showed no independent association with any anterior segment parameter; it was instead associated with cumulative glucocorticoid exposure (B = +1.602, *p* = 0.024). Conclusions: In CKD, clinical anterior segment and ocular surface differences were largely explained by age, sex, and comorbidity rather than renal replacement modality, with visual acuity deficit persisting in HD after adjusting for these covariates. At the individual level, corrected calcium was the parameter most consistently associated with tear film parameters in KTRs, whereas elevated Activin A was not independently associated with ocular involvement and likely reflects systemic disease activity.

## 1. Introduction

Chronic kidney disease (CKD) is a progressive systemic disorder characterized by irreversible decline in renal function, accompanied by metabolic, inflammatory, and vascular alterations that extend beyond the kidneys to multiple organ systems, including the eye [1,2]. The ocular circulation, particularly the retina and choroid, has been extensively studied in this context due to its structural and functional similarities to renal microvasculature, with numerous reports demonstrating retinal thinning, choroidal alterations, and microvascular rarefaction in CKD patients [1,3].

However, increasing evidence suggests that anterior segment structures are also significantly affected by the systemic stress characteristic of renal dysfunction. Uremic toxin accumulation, electrolyte imbalance, oxidative stress, and chronic low-grade inflammation may disrupt ocular surface homeostasis, impair lacrimal gland function, and alter corneal and lenticular physiology [4,5,6,7,8]. These mechanisms have been associated with a higher prevalence of dry eye disease, tear film instability, and epithelial alterations in patients with CKD, particularly in those undergoing hemodialysis (HD) [4,5,6,7].

In addition to chronic metabolic derangement, renal replacement therapies introduce further physiological stress. HD is associated with acute osmotic and hemodynamic fluctuations that can influence tear film composition, corneal hydration, and intraocular pressure (IOP) dynamics [8,9]. Conversely, ocular alterations may persist even in kidney transplant recipients (KTRs), despite partial restoration of renal function [10].

Cataract formation has also been increasingly linked to CKD, with epidemiological studies demonstrating a higher prevalence and earlier onset of lens opacification in patients with renal impairment [11,12]. These findings are thought to reflect cumulative oxidative stress, disturbances in calcium–phosphate metabolism, and the accumulation of advanced glycation end-products, all of which contribute to protein aggregation and lens degeneration [10,11,12].

Among the molecular mediators involved in CKD progression, Activin A has emerged as a regulator of inflammation, fibrosis, vascular remodeling, and mineral metabolism [13]. Elevated circulating Activin A levels have been reported in patients with advanced CKD and have been associated with cardiovascular and metabolic complications. However, little is known regarding the relationship between Activin A and ocular alterations in patients undergoing renal replacement therapy [14].

The present study aimed to investigate anterior segment findings across the spectrum of renal replacement therapy, including chronic HD and KTRs, and to assess their association with mineral metabolism and renal functional status.

## 2. Materials and Methods

### 2.1. Study Design

We conducted a cross-sectional comparative study with an interdisciplinary design, aimed at evaluating anterior segment alterations in patients with CKD and their relationship with renal function parameters and dialysis burden. A one-year prospective follow-up is planned.

Participants were recruited over a 12-month period and stratified into three predefined groups: patients undergoing chronic HD, KTRs, and a non-CKD reference group with preserved renal function.

Patients in the HD and KTR groups were consecutively recruited from individuals with CKD followed in the Nephrology Department. At the time of enrolment, all CKD participants underwent a comprehensive ophthalmological examination in the Ophthalmology Department, along with systemic evaluation, including renal and mineral metabolic profiling, as part of an interdisciplinary study protocol.

All participants provided written informed consent prior to inclusion, and the study adhered to the Declaration of Helsinki.

### 2.2. Study Population and Eligibility Criteria

Patients undergoing maintenance HD aged between 20 and 70 years, with a dialysis vintage of at least one year, were eligible for inclusion. No restrictions were applied regarding vascular access type or dialysis modality. To reduce the potential influence of acute hemodynamic changes, all ophthalmological assessments in the HD group were performed on a non-dialysis day.

KTRs aged 20–70 years with a stable, functioning graft for a minimum of one-year post-transplantation were also included. Eligibility was independent of donor type, prior dialysis exposure, or immunosuppressive regimen. In this group, clinical data collected included time since transplantation, duration of pre-transplant dialysis, type of immunosuppressive therapy, cumulative glucocorticoid exposure. CKD etiology (primary renal disease) was also collected in both groups.

A common set of exclusion criteria was applied to both patient groups to limit potential confounding factors. These comprised pregnancy or lactation, active treatment for osteoporosis (other than calcium and vitamin D supplementation), secondary causes of osteoporosis unrelated to CKD or transplantation, history of malignancy, liver cirrhosis, recent major fractures, prior non-renal organ transplantation, prior intraocular surgery (including cataract surgery and consequent pseudophakia), prior diagnosis of glaucoma, use of topical ocular medication, ongoing treatment for dry eye disease, contact lens wear and any active acute ocular or systemic illness at the time of evaluation. In addition, KTRs were excluded if they had experienced acute graft rejection or had returned to dialysis at the time of evaluation. Of 290 HD patients and 550 KTRs invited to participate, 29 and 48 were ultimately enrolled, respectively, after exclusion of those who did not meet the study criteria or declined to take part. Non-participation was attributable to declined consent or inability to fulfil the ophthalmological examination. No a priori sample-size calculation was performed; the study used a convenience sample of consenting eligible patients.

The non-CKD reference group was selected among patients referred by their general practitioner to the Endocrinology Department for routine evaluation, using the same general exclusion criteria. Additional exclusion criteria for this group included a diagnosis of CKD, diabetes mellitus (DM), or prolonged systemic corticosteroid therapy (defined as ≥7.5 mg/day prednisone equivalent for at least three months). Fifty-three non-CKD subjects were finally included in the study. Formal matching to the renal cohorts was not feasible in this single-center observational design; the control group therefore represents a non-CKD reference group rather than a strictly matched control population.

All participants underwent a comprehensive clinical evaluation during a single study visit. The assessment included a detailed medical history cross-checked against available medical records and a complete physical examination. Fasting morning blood samples for biochemical analysis were collected on the same day as the ophthalmological examination. The standardized ophthalmological evaluation for all participants included assessment of both the anterior and posterior segments. Posterior-segment structural and microvascular findings in the renal cohorts have been reported separately [15] and were not reanalyzed here; the present analysis focuses on best-corrected visual acuity (BCVA) and anterior-segment/ocular-surface parameters (detailed in Section 2.4).

### 2.3. Renal and Metabolic Assessment

Serum creatinine, calcium, and phosphate concentrations were determined using standard photometric/colorimetric methods. Serum intact PTH was measured by electrochemiluminescence immunoassay (ECLIA) and 25-hydroxyvitamin D by electrochemiluminescence binding assay, using a Roche cobas^®^ E601 immunoassay analyzer (Roche, Branchburg, NJ, USA). Serum Activin A concentrations were measured from fasting blood samples using a commercially available sandwich enzyme-linked immunosorbent assay (ELISA) kit (Human Activin A ELISA Kit, Cat. No. HUFI00027, Assay Genie Ltd., Dublin, Ireland) according to the manufacturer’s instructions.

Albumin-corrected calcium was calculated to account for variations in serum albumin concentration using the following formula: corrected calcium (mg/dL) = total calcium (mg/dL) + 0.8 × [4.0 − serum albumin (g/dL)] [16]. Renal function was assessed by calculating eGFR using the validated Chronic Kidney Disease Epidemiology Collaboration (CKD-EPI) equation [17].

### 2.4. Ophthalmologic Examination

All participants underwent a comprehensive ophthalmological evaluation during a single study visit, performed by the same experienced ophthalmologist under standardized examination conditions to minimize interobserver variability. The examination protocol included assessment of BCVA, tear film function, IOP, and detailed slit-lamp biomicroscopy (Topcon Corporation, Tokyo, Japan) of the anterior segment. BCVA was recorded as decimal acuity for each eye using standard clinical refraction protocols. Descriptive BCVA values are presented in decimal format for clinical readability. IOP was measured by Goldmann applanation tonometry (Topcon Corporation, Tokyo, Japan), with at least two consecutive measurements obtained for each eye and the mean value used for analysis.

Ocular surface and tear film evaluation included both the Schirmer I test and fluorescein TBUT. The Schirmer I test was performed without topical anesthesia under standardized conditions, and tear production was quantified based on the extent of strip wetting after 5 min. Schirmer I values ≥ 10 mm were considered within normal limits, while values < 10 mm were considered suggestive of reduced aqueous tear secretion. Tear film stability was assessed following fluorescein instillation, with TBUT defined as the interval between a complete blink and the first appearance of a dry spot on the corneal surface. For each eye, the mean of three consecutive TBUT measurements was considered for statistical analysis to improve measurement reproducibility. TBUT values ≥ 10 s were considered normal, whereas values < 10 s were interpreted as indicative of tear film instability.

Anterior segment examination was performed using slit-lamp biomicroscopy. Cataract was evaluated clinically based on the presence of lens opacification identified during examination and recorded as a binary variable (present/absent). Blepharitis was similarly documented as present or absent according to characteristic clinical findings, including eyelid margin hyperemia, crusting or scaling, and signs of meibomian gland dysfunction.

All ophthalmological examinations were completed on the same day, following a standardized sequence to minimize interference between tests: Best-corrected visual acuity (BCVA) assessment and slit-lamp biomicroscopy were performed first, as non-invasive procedures. Fluorescein TBUT was then measured, followed by the Schirmer I test, with a recovery interval of at least 5 min allowed between the two to permit tear-film stabilization and to limit the influence of fluorescein-induced reflex tearing on Schirmer values. IOP measurement using Goldmann applanation tonometry was performed last, ensuring that the topical anesthesia and fluorescein instillation required for applanation, as well as direct contact with the ocular surface, did not affect the preceding tear-film and ocular-surface assessments. A minimum interval of 5 min was maintained between examinations to allow tear-film recovery. Both eyes were examined in all participants, and ophthalmological parameters were recorded and analyzed separately for the right eye (RE) and left eye (LE).

### 2.5. Study Endpoints

The primary endpoint was the comparison of visual acuity and anterior segment parameters across the three study groups—patients on chronic HD, KTRs, and the non-CKD reference group. The secondary endpoint was the evaluation of the association between visual acuity and anterior segment findings and disease- and treatment-related factors within the CKD groups. In the HD group, this analysis focused on dialysis burden, assessed as dialysis vintage (duration of maintenance HD) and primary renal disease. In KTRs, it considered time since transplantation, duration of pre-transplant dialysis, cumulative glucocorticoid exposure, type of immunosuppressive regimen, eGFR and the primary indication for transplantation (underlying cause of end-stage renal disease).

The tertiary (exploratory) endpoint was the assessment of the relationship between visual acuity and anterior segment parameters and markers of mineral metabolism and systemic disease activity.

### 2.6. Statistical Analysis

Statistical analyses were performed using IBM SPSS Statistics for Macintosh (Version 29.0; IBM Corp., Armonk, NY, USA). Normality of the data distribution was assessed using the Shapiro–Wilk test, while homogeneity of variances was evaluated by Levene’s test when appropriate. Normally distributed continuous variables are presented as mean ± standard deviation (SD), whereas non-normally distributed variables are presented as median and interquartile range (IQR). Categorical variables are presented as counts and percentages.

Comparisons between two groups were conducted using the independent-sample Student’s *t*-test for normally distributed variables and the Mann–Whitney U test for variables with non-normal distribution. For comparisons among the three study groups, one-way analysis of variance (ANOVA) was applied. Post hoc pairwise analyses were performed using Tukey’s b test when variance homogeneity assumptions were met and the Games–Howell test when equal variances could not be assumed. For variables with non-normal distribution, the Kruskal–Wallis test was used. Categorical variables were compared using the chi-square test or Fisher’s exact test, as appropriate.

Relationships between continuous variables were explored using Pearson’s correlation coefficient for normally distributed data and Spearman’s rank correlation coefficient for non-parametric variables. Significant associations identified through correlation analysis were subsequently examined by bivariate linear regression, where appropriate, to further quantify the relationship between systemic and ophthalmological parameters. Missing data were handled by complete-case analysis; each model included only participants with complete data for the variables involved, which accounts for the differing sample sizes across analyses. The 25-hydroxyvitamin D was available in only 10 HD and 10 KTR patients and was therefore only analyzed descriptively.

For the adjusted analysis of anterior pole parameters, generalized estimating equation (GEE) models were used to account for inter-eye correlation, with both eyes included and clustered by patient. Models were adjusted for age, sex, body mass index (BMI) and DM status, and adjusted mean differences with 95% confidence intervals (CIs) were reported. Because decimal acuity is not linear, all correlation, regression, and adjusted GEE analyses involving visual acuity were performed using logMAR-transformed BCVA values, calculated as logMAR = −log10 (decimal acuity). Higher logMAR values indicate worse visual acuity. Additional sensitivity analyses were performed by including cataract status as an eye-level covariate in the logMAR BCVA model.

Cataract was analyzed as a binary eye-level outcome using binomial GEE models with logit link, exchangeable working correlation structure, and robust standard errors. Results are presented as adjusted odds ratios (ORs) with 95% CI. Activin A was analyzed as a patient-level variable; therefore, adjusted between-group comparisons of Activin A were performed using patient-level Gaussian linear models adjusted for age, sex, and diabetes status, without duplicating the same Activin A measurement for both eyes.

A two-sided *p*-value < 0.05 was considered statistically significant throughout the analysis. All reported *p*-values are nominal (uncorrected for multiple comparisons) given the exploratory, hypothesis-generating design and limited sample size.

## 3. Results

### 3.1. Descriptive Results

Baseline demographic, clinical, and biochemical characteristics of the study population are summarized in Table 1. KTRs were significantly younger than both HD patients and the reference group (49.0 [40.0–56.0] vs. 60.0 [50.0–68.0] and 57.0 [54.0–61.0] years, respectively; *p* < 0.001). Dialysis exposure was highly variable in the HD group, with a median dialysis vintage of 56.0 [12.0–132.0] months. In the KTR group, the median duration of pre-transplant dialysis was 4.5 [0.0–42.0] months, while the median time since transplantation was 83.5 [43.0–144.0] months, reflecting a predominantly long-term post-transplant population. All KTRs were receiving maintenance immunosuppressive therapy, typically consisting of calcineurin inhibitors (tacrolimus, 37/48; ciclosporin, 8/48), antiproliferative agents (mycophenolate mofetil, 47/48; azathioprine, 1/48), and corticosteroids (prednisone, 42/48), with a median cumulative glucocorticoid dose of 14.43 [9.93–24.90] g.

Alfacalcidol treatment was recorded in eight HD patients and 24 KTR patients, while calcium-based phosphate binders were used by 18 HD patients. None of the patients in the reference group were under supplementation with vitamin D or calcium.

The overall distribution of individual CKD etiologies (primary renal disease) as well as of broader clinical categories did not differ significantly between the HD and KTR groups (*p* = 0.366 and 0.056, respectively; Table 1).

BMI was significantly higher in the reference group compared with KTR patients (*p* = 0.030), while BCVA was generally comparable across groups. A modest but statistically significant reduction in BCVA was observed in the LE of HD patients compared with the reference group (*p* = 0.015; Table 1).

IOP values were within normal limits across all groups, with no statistically significant intergroup differences (all *p* > 0.05). Tear film parameters, including Schirmer I test values and TBUT, showed comparable mean values between groups at the descriptive level. Blepharitis prevalence was similar between groups. Cataract prevalence differed significantly among groups in the unadjusted descriptive analysis, being highest in the HD and lowest in the KTR group (Table 1).

Significant differences were observed in renal function parameters. As expected, KTRs exhibited higher serum creatinine levels and lower eGFR compared with the reference group (all *p* < 0.001), reflecting persistent renal impairment despite transplantation. HD patients demonstrated the most pronounced metabolic alterations, including higher serum phosphate levels compared with both non-CKD subjects and KTR patients, while corrected calcium values were lower in the HD than in the reference and KTR groups. Serum 25-hydroxyvitamin D levels did not differ significantly among groups (*p* > 0.05), although interpretation is limited by the smaller sample size (*N* = 10) in the renal cohorts (Table 1).

Circulating Activin A concentrations differed significantly among study groups. The highest levels were observed in HD patients, intermediate values in KTRs, and the lowest concentrations in controls (*p* < 0.001) (Table 1).

### 3.2. GEE-Adjusted Analysis

After adjustment for age, sex, BMI, and DM status, the overall group effect for logMAR BCVA was borderline (overall *p* = 0.055; Table 2). HD patients showed worse visual acuity than the reference group, reflected by higher logMAR BCVA values (adjusted B = +0.128 logMAR, 95% CI 0.017 to 0.240, *p* = 0.024), whereas KTRs did not differ significantly from either the reference or HD group. In a sensitivity analysis additionally adjusted for eye-level cataract status, the HD versus reference difference attenuated but remained significant (adjusted B = +0.107 logMAR, 95% CI 0.008 to 0.206, *p* = 0.033), while cataract status itself was associated with worse visual acuity (adjusted B = +0.142 logMAR, 95% CI 0.031 to 0.253, *p* = 0.012).

IOP showed no significant overall group effect, although HD patients had nominally higher IOP than the reference group (adjusted B = +1.31 mmHg, 95% CI 0.11 to 2.51, *p* = 0.032; Table 2). No significant adjusted differences were observed for TBUT or Schirmer I test values (Table 2).

After adjustment for age, sex, BMI and DM status, cataract prevalence no longer differed significantly between groups overall (overall *p* = 0.318). The adjusted odds of cataract were not significantly different for the HD group versus the reference group (OR = 1.8, 95% CI 0.64 to 5.09, *p* = 0.265), KTRs versus the reference group (OR = 0.67, 95% CI 0.25 to 1.78, *p* = 0.419), or KTRs versus the HD group (OR = 0.37, 95% CI 0.10 to 1.35, *p* = 0.132).

**Figure 1:**
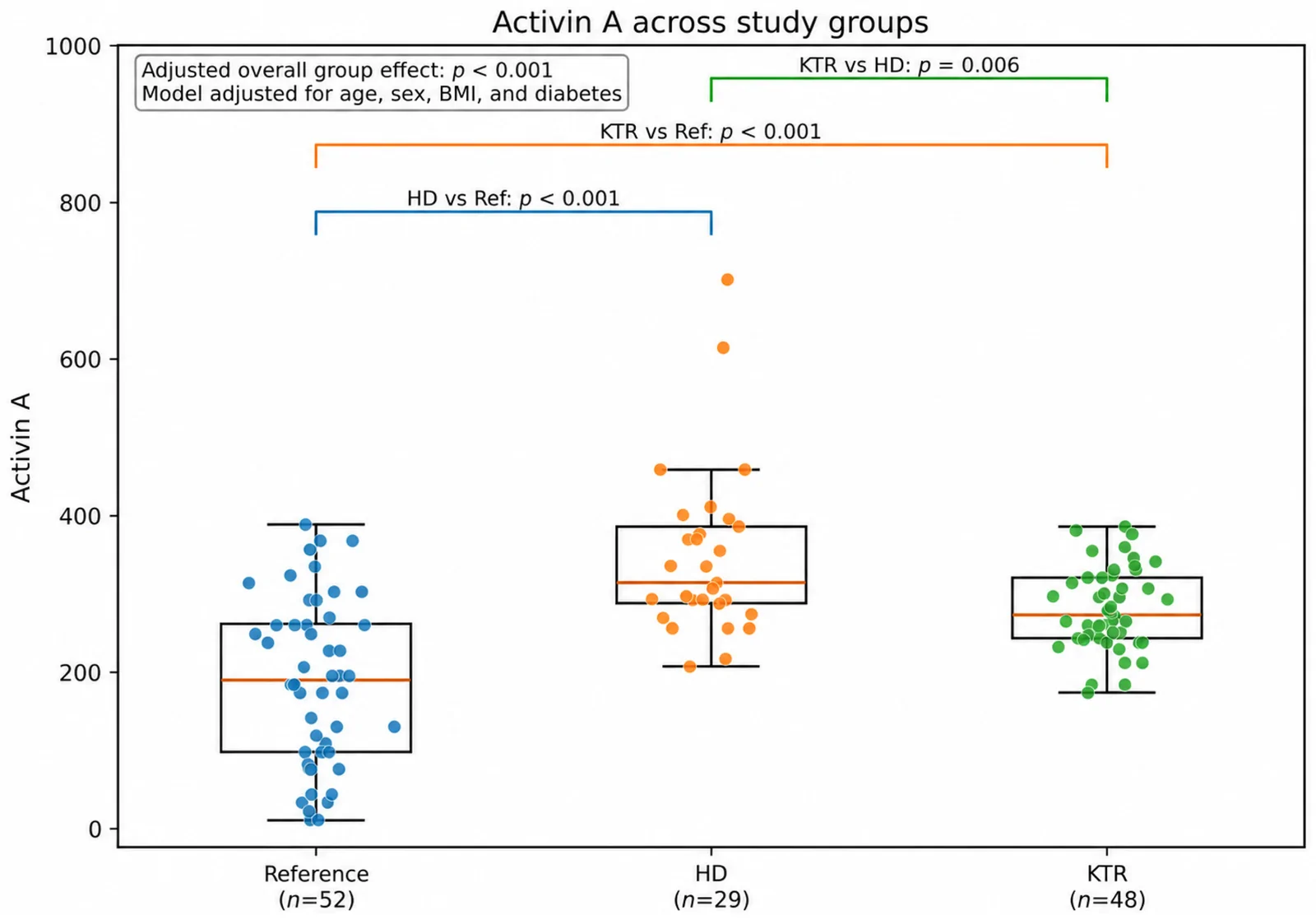
Adjusted comparison of Activin A levels across the study groups. Pairwise *p*-values were derived from patient-level Gaussian linear models adjusted for age, sex, BMI and diabetes status. Abbreviations: HD, hemodialysis; KTR, kidney transplant recipient; Ref, non-CKD reference group.

Activin A was analyzed separately as a patient-level variable using Gaussian linear models adjusted for age, sex, BMI and diabetes status, without duplicating values across eyes. After adjustment, Activin A levels were significantly higher in both the HD (adjusted B = +156.29, 95% CI 104.46 to 208.13, *p* < 0.001) and KTR groups (adjusted B = +85.46, 95% CI 46.75 to 124.17, *p* < 0.001) compared with the reference group and were significantly lower in KTRs than in the HD group (adjusted B = −70.83, 95% CI −121.01 to −20.66, *p* = 0.006; Figure 1).

### 3.3. Correlation Analysis

Correlation analyses were performed to investigate the relationships between visual acuity, anterior segment parameters and demographic, biochemical, and treatment-related variables. The significant correlations identified in each study group are summarized in Table 3; the complete set of correlation analyses, including all non-significant results, is provided in Supplementary Table S1.

In the reference group, older age was associated with worse visual acuity, reflected by positive correlations with logMAR BCVA in both eyes (RE: ρ = +0.418, *p* = 0.002; LE: ρ = +0.371, *p* = 0.006). Higher serum magnesium levels were associated with better visual acuity in the RE, reflected by a negative correlation with logMAR BCVA (ρ = −0.310, *p* = 0.024), while higher eGFR was associated with better visual acuity in the LE (ρ = −0.355, *p* = 0.009). In addition, IOP in the RE demonstrated a positive correlation with corrected calcium levels (ρ = 0.343, *p* = 0.013). A weak positive correlation was also observed between Activin A concentrations and TBUT in the RE (ρ = 0.334, *p* = 0.016) (Table 3).

Among HD patients, increasing age correlated with worse visual acuity in both eyes as shown by positive correlations with logMAR BCVA (RE: ρ = +0.389, *p* = 0.037; LE: ρ = +0.679, *p* < 0.001). Furthermore, TBUT in the RE demonstrated a moderate positive correlation with serum phosphate levels (ρ = 0.407, *p* = 0.029). No significant correlations were identified between dialysis vintage and anterior segment parameters.

In the KTR group, higher corrected calcium levels were associated with reduced tear film stability, reflected by a negative correlation with TBUT in the RE (ρ = −0.377, *p* = 0.008) and a similar trend for the LE. Schirmer I test values in the LE were also negatively correlated with corrected calcium levels (ρ = −0.317, *p* = 0.028). Longer pre-transplant dialysis duration was associated with worse visual acuity in both eyes, reflected by positive correlations with logMAR BCVA (RE: ρ = +0.384, *p* = 0.007; LE: ρ = +0.299, *p* = 0.039; Table 3).

In addition, Activin A concentrations were positively correlated with cumulative glucocorticoid dose (ρ = +0.337, *p* = 0.022) but showed no significant associations with anterior segment parameters.

### 3.4. Regression Analysis

When significant correlations were further explored using adjusted linear regression models, only selected associations remained significant after covariate adjustment. Models were adjusted for age, sex, BMI and DM status when applicable; when age was the predictor of interest, models were adjusted for sex, BMI and DM status only. Results are depicted in Table 4.

In linear regression models adjusted for the predefined covariate set, including age, sex, BMI, and DM status, age was the strongest contributor to worse visual acuity in the LE in the HD group, reflected by higher logMAR BCVA values (standardized β = +0.402, *p* = 0.021; R^2^ = 0.395). In KTRs, corrected calcium contributed independently to lower Schirmer I LE and TBUT RE values (β = −0.349, *p* = 0.008, R^2^ = 0.141 and β = −0.318, *p* = 0.037, R^2^ = 0.135, respectively), while cumulative glucocorticoid dose was positively associated with Activin A (β = +0.341, *p* = 0.024; R^2^ = 0.163). In the reference group, age contributed to worse logMAR BCVA in the RE (β = +0.348, *p* = 0.005; R^2^ = 0.193), whereas Activin A was positively associated with TBUT RE (β = +0.314, *p* = 0.041; R^2^ = 0.124).

### 3.5. Additional Exploratory Analyses

Additional exploratory analyses were performed to assess whether mineral metabolism markers differed according to cataract status or tear film abnormalities. In the overall cohort, participants with cataract had higher serum phosphate concentrations than those without cataract (3.96 ± 1.52 vs. 3.38 ± 0.88 mg/dL, *p* = 0.044), whereas corrected calcium, magnesium, PTH, and 25-OH vitamin D concentrations did not differ significantly. No significant differences in corrected calcium, phosphate, magnesium, PTH, or 25-OH vitamin D were observed between participants with and without tear film abnormalities (all *p* > 0.05).

Exploratory correlation analyses were also performed to assess the relationship between medication use and anterior pole parameters within the HD and KTR groups. No significant correlations were observed between alfacalcidol or calcium-based phosphate binder use and BCVA, IOP, TBUT, or Schirmer test values in either group. In KTRs, alfacalcidol use was positively associated with cataract in the right eye (ρ = +0.396, *p* = 0.005).

## 4. Discussion

This study yielded several observations regarding visual acuity and anterior segment findings in renal replacement therapy. After adjustment for age, sex, BMI and DM, HD patients retained worse visual acuity than the reference group, whereas the higher cataract prevalence seen at the descriptive level no longer differed significantly between groups. Higher corrected calcium was independently associated with reduced tear film stability in KTRs. Finally, circulating Activin A, although it differed significantly across the renal-disease spectrum, showed no independent association with anterior segment parameters; instead, it was linked to cumulative glucocorticoid exposure in KTRs, and in the reference group, to a higher TBUT.

### 4.1. Visual Acuity in HD Group

The most consistent group-level finding was that HD patients retained worse visual acuity than the reference group after adjustment for age, sex, BMI and DM, indicating a deficit not fully explained by these covariates. In the absence of cornea, retina, and optic nerve data, this finding is best interpreted as a difference in overall visual acuity rather than a specific anterior-segment alteration. Importantly, the HD–reference difference in visual acuity persisted after additional adjustment for cataract status, suggesting that the deficit is not explained by lens status alone and pointing to a contribution beyond the anterior segment. Several mechanisms characteristic of the HD state may plausibly contribute to this, including acute osmotic and hemodynamic fluctuations that transiently alter corneal hydration and refraction [7], subclinical lens changes below the threshold of clinical cataract grading [18,19], and uremia-related microvascular injury affecting the posterior segment [1,2,3,4,5]. The independent bilateral finding is more consistent with a systemic contribution than with an isolated ocular cause, although the cross-sectional design and the absence of cornea and posterior-segment data preclude firm mechanistic conclusions.

Within the HD group, lower visual acuity was associated with older age but not with HD vintage. This argues against a simple cumulative-exposure model, in which longer time on HD would be expected to track with progressive visual deterioration, and instead points to age-related ocular changes as the dominant contributor at the individual level.

Comparable inconsistency exists in the literature: longer HD exposure has been linked to structural ocular changes such as conjunctival and corneal calcification and cataract [20], yet visual acuity in these cohorts is often driven more by age, diabetic retinopathy, and comorbidity than by dialysis duration [21], and mineral-metabolic associations with ocular findings that appear in univariate analysis frequently do not survive multivariable adjustment [21]. Acute intradialytic shifts in refraction and visual acuity have also been documented, underscoring the dynamic influence of the hemodialysis state on the eye [22]. Nonetheless, our findings support periodic ophthalmologic assessment in patients on maintenance HD.

### 4.2. Cataract

Cataract was more prevalent in HD than in KTRs at the descriptive level, but this difference did not persist after adjustment for age, sex, BMI and comorbidity, indicating that this difference was largely driven by the older age of the HD group rather than by renal status itself.

Nonetheless, the crystalline lens is an avascular structure dependent on tightly regulated metabolic homeostasis, and chronic exposure to uremic toxins, oxidative stress, and calcium–phosphate disturbances has been implicated in lens protein oxidation, disruption of fiber architecture, and accelerated lens aging [18,19]. Participants with cataract in our cohort had higher serum phosphate concentrations. However, this association was unadjusted, and it may largely reflect between-group differences—with cataract being more frequent in the HD cohort, the group with the highest phosphate—rather than a specific lenticular effect. It should therefore be interpreted with caution.

In exploratory analyses, alfacalcidol use in KTRs was positively associated with cataract in the RE but not the left. Because this association was not mirrored bilaterally, it should be interpreted with caution and may reflect chance, residual confounding, or the exploratory nature of the analysis.

### 4.3. Blepharitis

Blepharitis prevalence did not differ across groups despite marked differences in renal and mineral-metabolic status, suggesting that clinically apparent eyelid inflammation is driven more by local factors—e.g., microbial colonization, meibomian gland dysfunction—than by systemic disease severity [12]. This does not exclude subclinical inflammation, which may require tear biomarkers or detailed meibomian gland assessment for detection.

### 4.4. Tear Film Particularities

Schirmer I and TBUT values did not differ significantly among groups, contrasting with reports of increased features of dry eye disease in renal impairment. Aktaş et al. [10] and Türkcü et al. [11] described reduced tear secretion, shorter break-up times, and corneal alterations in CKD, attributed to chronic inflammation, oxidative stress, autonomic dysfunction, and meibomian gland changes [10,11]. The findings are inconsistent, however; Asiedu et al. [23] found no effect of CKD on tear film substance P in type 2 DM, and differences in CKD severity, dialysis exposure, and methodology likely explain such discrepancies [23].

In our cohort, the apparent preservation of tear film parameters at the group level suggests that global clinical measures such as Schirmer I and TBUT may be too insensitive to capture subtle ocular surface changes rather than reflecting a true absence of systemic influence. Supporting this, regression analysis in KTRs pointed towards calcium as a candidate modulator of tear film stability (see below).

### 4.5. Corrected Calcium and Tear Film Stability in KTRs

In KTRs, higher corrected calcium was independently associated with reduced tear film stability, correlating with both shorter TBUT and lower Schirmer I values after adjustment for age, sex, BMI and DM status. This inverse relationship is biologically plausible: calcium is essential to lacrimal acinar secretion and to the integrity of ocular surface epithelial junctions, and disturbances in calcium homeostasis may impair both aqueous tear production and tear film stability [24,25]. In KTRs—in whom mineral metabolism is influenced by residual hyperparathyroidism, immunosuppressive therapy, and variable graft function—even modest increases in corrected calcium may therefore have measurable effects on the ocular surface. Notably, alfacalcidol treatment was not correlated with TBUT or Schirmer I values in KTRs; while the calcium–tear film relationship apparently tracks the circulating calcium level itself, the finding is exploratory and requires confirmation in follow-up studies.

### 4.6. Activin A and Systemic Disease Burden

An additional finding of this study was the marked increase in circulating Activin A levels across the spectrum of renal replacement therapy. Activin A concentrations were lowest in the reference group, intermediate in KTRs, and highest in patients undergoing HD, suggesting a graded relationship with CKD severity and cumulative systemic disease burden, after adjusting for age and sex.

Activin A is a TGF-β superfamily cytokine linked to chronic inflammation, tissue fibrosis, vascular remodeling, endothelial dysfunction, and disturbances in mineral and bone metabolism [26,27,28,29]. In CKD, it drives the osteogenic transition of vascular smooth muscle cells and has been implicated in vascular calcification and cardiovascular disease, positioning it as a marker of systemic cardiovascular risk [30].

In our study, levels remained elevated in KTRs despite restored renal function, likely reflecting incomplete reversal of CKD-associated vascular and inflammatory alterations after transplantation. Activin A was not independently associated with any anterior segment parameter, apart from a weak positive association with TBUT in the reference group of uncertain significance—suggesting it marks systemic disease activity rather than directly determining ocular change. Given its vascular and profibrotic profile, it may instead relate to the retinal and choroidal microvasculature; this is being evaluated separately.

Activin A was also independently associated with cumulative glucocorticoid exposure in KTRs. This most likely reflects confounding by cumulative disease and inflammatory burden rather than a direct pharmacological effect, as greater cumulative glucocorticoid exposure tends to accompany a more complex post-transplant evolution. A contributory biological interaction cannot be excluded, since glucocorticoids influence bone remodeling and TGF-β/activin-family signaling, and Activin A itself regulates osteoclastogenesis in CKD–mineral bone disorder [27]. However, because we measured neither cardiovascular outcomes nor a composite index of disease burden, this association is exploratory and warrants confirmation.

### 4.7. Strengths and Limitations

To our knowledge, this is the first study to evaluate mineral metabolism, circulating Activin A, and anterior segment characteristics across renal replacement modalities, alongside a non-CKD reference group. Several limitations should nonetheless be acknowledged. The cross-sectional design precludes causal inference. Schirmer I and TBUT are inherently variable and may miss subclinical ocular surface changes, and cataract and blepharitis were recorded without detailed grading, limiting analysis by severity. The low participation rate raises the possibility of selection bias, and the convenience-sampling design further limits generalizability. The limited sample size, particularly within subgroups, reduced power and increased type II error risk in exploratory analyses; because candidate associations for regression modeling were selected from significant univariate correlations and *p*-values were not corrected for multiple comparisons, the reported associations may be subject to false-positive or over-estimated effects. The 25-hydroxyvitamin D data were available in only a subset of participants.

Regarding cohort comparability, diabetes was an exclusion criterion in the reference group but present in a small subset of HD and KTR patients, and the KTR group was significantly younger than the other groups; although diabetes status and age were included as covariates in all adjusted models, residual confounding from these differences cannot be completely excluded. Finally, retinal status was documented as part of the comprehensive ophthalmological evaluation, and the posterior-segment findings in the renal cohorts were reported separately [15]; these were not incorporated into the present analyses, which focused specifically on anterior-segment outcomes. Coexisting retinal pathology may therefore contribute to BCVA, and residual confounding by this factor cannot be excluded. The relationship of Activin A to the posterior segment is under separate investigation. These findings are hypothesis-generating and require confirmation in larger, longitudinal cohorts.

## 5. Conclusions

This study shows that HD was associated with a residual reduction in visual acuity that persisted after adjustment for age, sex, BMI and DM, whereas most other differences reflected age and comorbidity rather than renal replacement therapy—notably the higher cataract prevalence in the HD group, which disappeared after adjustment. Higher corrected calcium was independently associated tear film abnormalities (shorter TBUT and lower Schirmer I values) in KTRs, whereas circulating Activin A—although progressively elevated from non-CKD to transplant recipients to HD patients—showed no independent association with any anterior segment parameter. These associations are exploratory and require confirmation in larger longitudinal cohorts.

## Figures and Tables

**Table 1:** Baseline demographic, ophthalmological, and biochemical characteristics of the study groups.

Variable	Reference Group (*n* = 53)	HD (*n* = 29)	KTR (*n* = 48)	*p*-Value
Age (years)	57.00 [54.00–61.00] ^b^	60.00 [50.00–68.00] ^b^	49.00 [40.00–56.00] ^ac^	<0.001
Female sex, *n* (%)	42 (79.2%) ^ab^	15 (51.7%) ^c^	24 (50.0%) ^c^	0.004
BMI (kg/m^2^)	29.92 ± 5.08 ^b^	27.62 ± 4.24	27.61 ± 4.87 ^c^	0.030
BCVA RE	1.00 [0.90–1.00]	0.90 [0.80–1.00]	1.00 [0.90–1.00]	0.130
BCVA LE	1.00 [0.90–1.00] ^a^	0.90 [0.70–1.00] ^c^	1.00 [0.88–1.00]	0.015
IOP RE (mmHg)	15.00 [12.00–17.00]	16.00 [15.00–18.00]	16.00 [12.75–17.00]	0.097
IOP LE (mmHg)	16.00 [13.00–17.00]	17.00 [15.00–17.00]	16.00 [12.00–17.00]	0.465
TBUT RE (s)	10.00 [7.00–10.00]	10.00 [7.00–11.00]	10.00 [9.00–11.00]	0.086
TBUT LE (s)	10.00 [8.00–11.00]	10.00 [8.00–11.00]	10.00 [9.75–11.00]	0.332
Schirmer I RE (mm)	12.00 [7.00–20.00]	12.50 [7.00–18.50]	10.00 [7.00–20.25]	0.843
Schirmer I LE (mm)	12.00 [7.00–20.00]	15.50 [7.75–20.00]	15.00 [9.00–27.75]	0.193
Cataract RE, *n* (%)	17 (32.1%) ^a^	14 (48.3%) ^a^	7 (14.6%) ^c^	0.006
Cataract LE, *n* (%)	18 (34.0%) ^a^	13 (44.8%) ^a^	7 (14.6%) ^c^	0.011
Blepharitis, *n* (%)	10 (18.9%)	1 (3.4%)	8 (16.7%)	0.148
Cataract presence in either eye, *n* (%) †	19/53 (35.8%)	16/29 (55.2%) ^b^	9/48 (18.8%) ^a^	0.004
Tear film abnormalities, *n* (%) ‡	32/53 (60.4%)	17/29 (58.6%)	20/48 (41.7%)	0.135
DM status, *n* (%)	0 (0.0)	4 (13.8)	7 (14.6)	0.053
Creatinine (mg/dL)	0.74 ± 0.15 ^b^	—	1.51 ± 0.65 ^c^	<0.001
eGFR (mL/min/1.73 m^2^)	100.00 [91.00–104.00] ^b^	—	59.00 [45.50–70.12] ^c^	<0.001
Calcium (mg/dL)	9.05 [8.70–9.40] ^a^	8.50 [8.30–9.10] ^bc^	8.90 [8.78–9.30] ^a^	0.002
Phosphate (mg/dL)	3.40 [3.02–3.68] ^ab^	5.00 [4.30–5.40] ^bc^	2.95 [2.50–3.30] ^ac^	<0.001
PTH (pg/mL)	48.30 [38.45–62.25] ^ab^	303.00 [133.00–557.00] ^bc^	78.35 [51.18–101.83] ^ac^	<0.001
Magnesium (mg/dL)	2.00 [1.90–2.10] ^ab^	2.10 [2.00–2.40] ^bc^	1.70 [1.50–1.80] ^ac^	<0.001
Activin A (pg/mL)	189.50 [98.00–262.50] ^ab^	314.00 [288.00–386.00] ^bc^	273.00 [243.00–321.00] ^ac^	<0.001
25-OH Vitamin D (ng/mL)	29.51 ± 10.54	29.50 ± 15.58 (*n* = 10)	26.58 ± 11.37 (*n* = 10)	0.789
Dialysis vintage (months)	—	56.00 [12.00–132.00]	4.50 [0.00–42.00]	<0.001
Time since transplant (months) *	—	—	83.50 [43.00–144.00]	—
Cumulative glucocorticoid dose (g)	—	—	14.43 [9.93–24.90]	—
Primary renal etiology, *n* (%)				0.056
Unknown/ unspecified	—	8 (27.6%)	17 (35.4%)	
Glomerular/immune	—	6 (20.7%)	11 (22.9%)	
Congenital/hereditary	—	5 (17.2%)	11 (22.9%)	
Vascular/metabolic	—	8 (27.6%)	2 (4.2%)	
Tubulointerstitial/other	—	2 (6.9%)	7 (14.6%)	

Data are presented as mean ± SD for normally distributed variables, median [IQR] for non-normally distributed variables, or n (%), as appropriate. —, not applicable. * Duration of pre-transplant dialysis in kidney transplant recipients (KTRs). The *p*-value for sex distribution was calculated using Pearson’s chi-square test. Primary renal etiologies were grouped into five categories: unknown/unspecified, glomerular/immune, congenital/hereditary, vascular/metabolic, and tubulointerstitial/other. † Cataract was defined as the presence of cataract in either eye. ‡ Tear-film abnormalities were defined as TBUT < 10 s (tear-film instability) and/or Schirmer I test < 10 mm (reduced aqueous tear secretion) in either eye. Superscript letters indicate significant pairwise differences following post hoc analysis: a, significantly different from the HD group; b, significantly different from the KTR group; c, significantly different from the reference group (*p* < 0.05). Abbreviations: BCVA, best-corrected visual acuity; BMI, body mass index; CKD, chronic kidney disease; DM, diabetes mellitus; eGFR, estimated glomerular filtration rate; HD, hemodialysis; IOP, intraocular pressure; KTR, kidney transplant recipient; LE, left eye; PTH, parathyroid hormone; RE, right eye; SD, standard deviation; TBUT, tear film break-up time.

**Table 2:** GEE age-, sex-, BMI- and diabetes-adjusted comparisons of visual acuity and anterior segment parameters across study groups.

Outcome	Overall Group *p*	Contrast	Adjusted B	95% CI	*p*-Value
logMAR BCVA	0.055	HD vs. Reference	+0.128	0.017 to 0.240	0.024
		KTR vs. Reference	−0.01	−0.058 to 0.038	0.685
		KTR vs. HD	−0.138	−0.28 to 0.004	0.057
IOP, mmHg	0.101	HD vs. Reference	+1.31	0.11 to 2.51	0.032
		KTR vs. Reference	+0.64	−0.69 to 1.97	0.344
		KTR vs. HD	−0.67	−1.99 to 0.65	0.321
TBUT, s	0.803	HD vs. Reference	+0.01	−0.96 to 0.97	0.987
		KTR vs. Reference	+0.24	−0.53 to 1	0.542
		KTR vs. HD	+0.23	−0.72 to 1.18	0.635
Schirmer I test, mm	0.880	HD vs. Reference	+0.32	−3.62 to 4.26	0.874
		KTR vs. Reference	+0.9	−2.62 to 4.43	0.615
		KTR vs. HD	+0.58	−3.46 to 4.63	0.777

Values are adjusted mean differences from Gaussian generalized estimating equation models adjusted for age, sex, BMI and diabetes status. Positive B values indicate higher values in the first group listed in the contrast. Abbreviations: BCVA, best-corrected visual acuity; logMAR, logarithm of the minimum angle of resolution; BMI, body mass index; IOP, intraocular pressure; TBUT, tear break-up time; HD, hemodialysis; KTR, kidney transplant recipient; CI, confidence interval.

**Table 3:** Significant correlations between ocular parameters and systemic variables according to study group.

Group	Anterior Segment Parameter	Systemic Variable	Correlation Coefficient (ρ)	*p*-Value
Reference group	IOP RE	Corrected calcium	0.343	0.013
	logMAR BCVA RE	Age	+0.418	0.002
		Magnesium	−0.310	0.024
	logMAR BCVA LE	Age	+0.371	0.006
		eGFR	−0.355	0.009
	TBUT RE	Activin A	0.334	0.016
HD	TBUT RE	Phosphate	0.407	0.029
	logMAR BCVA RE	Age	+0.389	0.037
	logMAR BCVA LE		+0.679	<0.001
KTR	TBUT RE	Corrected calcium	−0.377	0.008
	logMAR BCVA RE	Pre-transplant dialysis duration	+0.384	0.007
	logMAR BCVA LE		+0.299	0.039
	Schirmer I LE	Corrected calcium	−0.317	0.028

Only statistically significant correlations (*p* < 0.05) between ocular parameters and systemic variables are shown. Abbreviations: BCVA, best-corrected visual acuity; logMAR, logarithm of the minimum angle of resolution; eGFR, estimated glomerular filtration rate; HD, hemodialysis; IOP, intraocular pressure; KTR, kidney transplant recipient; LE, left eye; RE, right eye; TBUT, tear break-up time.

**Table 4:** Adjusted regression analysis of visual acuity and anterior pole—systemic associations identified in exploratory correlation analyses.

Group	Dependent Variable	Predictor	*N*	Adjusted B	95% CI	*p*-Value	Standardized β	R^2^
Reference group	IOP RE	Corrected calcium	52	+1.675	−0.022 to 3.576	0.084	+0.266	0.159
	logMAR BCVA RE	Age	53	+0.0034	0.0011 to 0.0057	0.005	0.348	0.193
	logMAR BCVA RE	Magnesium		−0.129	−0.267 to 0.009	0.066	−0.332	0.288
	logMAR BCVA LE	Age		0.0020	−0.0003 to 0.0043	0.084	0.266	0.123
	logMAR BCVA LE	eGFR		−0.0002	−0.0016 to 0.0012	0.747	−0.061	0.127
	TBUT RE	Activin A	52	+0.0060	0.0003 to 0.0117	0.041	+0.314	0.124
HD	TBUT RE	Phosphate	29	+0.779	−0.570 to 2.128	0.258	+0.466	0.192
	logMAR BCVA RE	Age		0.0082	−0.013 to 0.0294	0.431	0.149	0.181
	logMAR BCVA LE			0.0047	0.0008 to 0.0086	0.021	0.402	0.395
KTR	TBUT RE	Corrected calcium	48	−0.903	−1.750 to −0.057	0.037	−0.318	0.135
	logMAR BCVA RE	Pre-transplant dialysis duration		0.00065	−0.00079 to 0.0021	0.369	0.271	0.215
	BCVA LE			0.00081	−0.0015 to 0.0032	0.492	0.182	0.211
	Schirmer I LE	Corrected calcium		−5.947	−10.356 to −1.539	0.008	−0.349	0.141
	Activin A	Cumulative glucocorticoid dose	46	+1.602	0.213 to 2.991	0.024	+0.341	0.163

Values are adjusted regression coefficients from linear regression models performed within each study group. The anterior pole parameter was entered as the dependent variable, and the corresponding systemic or treatment-related variable was entered as the predictor of interest. All models were adjusted for age, sex, BMI, and diabetes status; when age was the predictor of interest, models were adjusted for sex, diabetes status, and BMI only. In the reference group, diabetes status was not included because no participants had DM. Adjusted B values indicate the expected change in the dependent variable for a one-unit increase in the predictor while holding the other covariates constant. Abbreviations: BCVA, best-corrected visual acuity; logMAR, logarithm of the minimum angle of resolution; CI, confidence interval; eGFR, estimated glomerular filtration rate; HD, hemodialysis; IOP, intraocular pressure; KTR, kidney transplant recipient; LE, left eye; RE, right eye; R^2^, coefficient of determination; TBUT, tear break-up time.

## Data Availability

The original contributions presented in this study are included in the article/Supplementary Materials. Further inquiries can be directed to the corresponding author.

## Supplementary Materials

The following supporting information can be downloaded at https://www.mdpi.com/article/10.3390/life16081294/s1, Table S1: Complete exploratory correlation analysis.

## Abbreviations

The following abbreviations are used in this manuscript:

ANOVA	One-way analysis of variance
BCVA	Best corrected visual acuity
BMI	Body mass index
CI	Confidence interval
CKD	Chronic kidney disease
CKD-EPI	Chronic Kidney Disease Epidemiology Collaboration
CKD-MBD	CKD-related mineral and bone disorder
DM	Diabetes mellitus
ECLIA	Electrochemiluminescence immunoassay
eGFR	Estimated glomerular filtration rate
ELISA	Enzyme-linked immunosorbent assay
GEE	Generalized estimating equation
HD	Hemodialysis
ICC	Intraclass correlation coefficient
IOP	Intraocular pressure
KTR	Kidney transplant recipient
LE	Left eye
OR	Odds ratio
PTH	Parathyroid hormone
RE	Right eye
SD	Standard deviation
TBUT	Tear break-up time
TGF-β	Transforming growth factor beta
