# Supplementary material for: Clinical Anterior Segment and Ocular Surface Findings Across Renal Replacement Modalities: Associations with CKD-Related Factors, Mineral Metabolism and Activin A
Source: Life (Basel). 2026 Aug 6;16(8):1294. doi: 10.3390/life16081294 (PMC13514610; doi:10.3390/life16081294)
Supplement: Supplementary file 1 [file life-16-01294-s001.zip › life-4470653-supplementary.pdf]

Supplementary Table S1. Complete exploratory correlation analysis

Group	Parameter	Systemic/treatment-related variable	N	Spearman rho (ρ)	Nominal p-value
Reference group	logMAR BCVA RE	Age	53	+0.418	0.0018
		BMI		+0.119	0.3979
		Corrected calcium	52	-0.074	0.6039
		Phosphate	46	+0.003	0.9858
		PTH	50	-0.037	0.7994
		Magnesium	53	-0.310	0.0240
		Activin A	52	-0.134	0.3436
		Creatinine	53	+0.139	0.3193
		eGFR		-0.173	0.2147
	logMAR BCVA LE	Age	53	+0.371	0.0062
		BMI		+0.120	0.3924
		Corrected calcium	52	+0.125	0.3774
		Phosphate	46	-0.045	0.7680
		PTH	50	+0.006	0.9671
		Magnesium	53	-0.173	0.2160
		Activin A	52	-0.138	0.3277
		Creatinine	53	+0.256	0.0641
		eGFR		-0.355	0.0090
	IOP RE	Age	53	+0.026	0.8533
		BMI		+0.085	0.5431
		Corrected calcium	52	+0.343	0.0128
		Phosphate	46	+0.058	0.7004
		PTH	50	+0.239	0.0952
		Magnesium	53	-0.139	0.3197
		Activin A	52	+0.087	0.5377
		Creatinine	53	-0.031	0.8278
		eGFR		-0.069	0.6218
	IOP LE	Age	53	-0.044	0.7520
		BMI		+0.135	0.3352
		Corrected calcium	52	+0.118	0.4028
		Phosphate	46	+0.015	0.9218
		PTH	50	+0.145	0.3150
		Magnesium	53	+0.026	0.8511
		Activin A	52	+0.129	0.3614
		Creatinine	53	-0.035	0.8028

Group	Parameter	Systemic/treatment-related variable	N	Spearman rho (ρ)	Nominal p-value
		eGFR		-0.019	0.8926
	TBUT RE	Age	53	-0.106	0.4479
		BMI		-0.001	0.9964
		Corrected calcium	52	+0.052	0.7126
		Phosphate	46	-0.253	0.0902
		PTH	50	-0.053	0.7122
		Magnesium	53	-0.071	0.6130
		Activin A	52	+0.334	0.0156
		Creatinine	53	+0.038	0.7897
		eGFR		+0.002	0.9860
	TBUT LE	Age	53	-0.063	0.6564
		BMI		-0.097	0.4892
		Corrected calcium	52	-0.064	0.6528
		Phosphate	46	-0.201	0.1811
		PTH	50	+0.066	0.6497
		Magnesium	53	-0.034	0.8094
		Activin A	52	+0.132	0.3521
		Creatinine	53	-0.092	0.5113
		eGFR		+0.120	0.3909
	Schirmer I RE	Age	53	+0.046	0.7456
		BMI		-0.010	0.9419
		Corrected calcium	52	+0.095	0.5034
		Phosphate	46	-0.095	0.5296
		PTH	50	-0.135	0.3504
		Magnesium	53	-0.025	0.8609
		Activin A	52	+0.172	0.2222
		Creatinine	53	+0.134	0.3392
		eGFR		-0.104	0.4567
	Schirmer I LE	Age	53	+0.070	0.6181
		BMI		-0.084	0.5494
		Corrected calcium	52	+0.182	0.1971
		Phosphate	46	-0.083	0.5829
		PTH	50	-0.114	0.4317
		Magnesium	53	+0.035	0.8044
		Activin A	52	+0.169	0.2313
		Creatinine	53	-0.062	0.6580
		eGFR		+0.068	0.6292
**HD**	logMAR BCVA RE	Age	29	+0.389	0.0370
		BMI		+0.169	0.3800
		Corrected calcium		-0.108	0.5756

Group	Parameter	Systemic/treatment-related variable	N	Spearman rho (ρ)	Nominal p-value
		Phosphate		+0.041	0.8341
		PTH		-0.155	0.4220
		Magnesium		-0.001	0.9946
		Activin A		-0.360	0.0550
		Dialysis vintage		+0.178	0.3567
	logMAR BCVA LE	Age	29	+0.679	<0.001
		BMI		+0.242	0.2053
		Corrected calcium		-0.248	0.1939
		Phosphate		+0.054	0.7805
		PTH		-0.087	0.6551
		Magnesium		-0.163	0.3975
		Activin A		-0.301	0.1126
		Dialysis vintage		+0.043	0.8254
	IOP RE	Age	29	+0.064	0.7408
		BMI		+0.233	0.2248
		Corrected calcium		-0.032	0.8687
		Phosphate		+0.064	0.7430
		PTH		+0.113	0.5602
		Magnesium		+0.098	0.6140
		Activin A		+0.041	0.8308
		Dialysis vintage		-0.104	0.5917
	IOP LE	Age	29	+0.091	0.6391
		BMI		+0.176	0.3607
		Corrected calcium		+0.020	0.9183
		Phosphate		+0.296	0.1189
		PTH		+0.018	0.9242
		Magnesium		+0.140	0.4684
		Activin A		-0.063	0.7444
		Dialysis vintage		-0.198	0.3038
	TBUT RE	Age	29	-0.124	0.5223
		BMI		+0.034	0.8607
		Corrected calcium		-0.260	0.1726
		Phosphate		+0.407	0.0285
		PTH		-0.283	0.1373
		Magnesium		+0.155	0.4232
		Activin A		-0.043	0.8256
		Dialysis vintage		-0.275	0.1484
	TBUT LE	Age	29	-0.215	0.2630
		BMI		-0.025	0.8973
		Corrected calcium		-0.225	0.2399

Group	Parameter	Systemic/treatment-related variable	N	Spearman rho (ρ)	Nominal p-value
		Phosphate		+0.273	0.1519
		PTH		-0.005	0.9815
		Magnesium		+0.129	0.5051
		Activin A		-0.084	0.6660
		Dialysis vintage		-0.188	0.3295
	Schirmer I RE	Age	28	-0.067	0.7344
		BMI		+0.256	0.1894
		Corrected calcium		+0.043	0.8287
		Phosphate		+0.145	0.4624
		PTH		-0.115	0.5598
		Magnesium		+0.229	0.2402
		Activin A		-0.004	0.9850
		Dialysis vintage		+0.002	0.9928
	Schirmer I LE	Age	28	-0.167	0.3951
		BMI		+0.192	0.3287
		Corrected calcium		-0.041	0.8355
		Phosphate		-0.034	0.8646
		PTH		+0.057	0.7715
		Magnesium		+0.023	0.9065
		Activin A		-0.015	0.9380
		Dialysis vintage		-0.018	0.9286
**KTR**	logMAR BCVA RE	Age	48	+0.013	0.9279
		BMI		-0.047	0.7507
		Corrected calcium		-0.169	0.2515
		Phosphate		-0.147	0.3204
		PTH		+0.034	0.8179
		Magnesium	47	-0.057	0.7040
		Activin A	48	+0.009	0.9542
		Creatinine		-0.135	0.3612
		eGFR		+0.022	0.8803
		Pre-transplant dialysis duration		+0.384	0.0071
		Time since transplant		+0.084	0.5686
		Cumulative glucocorticoid dose	46	+0.129	0.3926
	logMAR BCVA LE	Age	48	+0.126	0.3922
		BMI		-0.108	0.4652
		Corrected calcium		-0.124	0.4003
		Phosphate		-0.079	0.5949
		PTH		+0.156	0.2893
		Magnesium	47	-0.056	0.7100
		Activin A	48	-0.130	0.3793

Group	Parameter	Systemic/treatment-related variable	N	Spearman rho (ρ)	Nominal p-value
		Creatinine		-0.156	0.2906
		eGFR		+0.005	0.9754
		Pre-transplant dialysis duration		+0.299	0.0393
		Time since transplant		+0.038	0.7951
		Cumulative glucocorticoid dose	46	-0.046	0.7612
	IOP RE	Age	48	+0.072	0.6249
		BMI		-0.022	0.8818
		Corrected calcium		-0.019	0.8973
		Phosphate		+0.143	0.3311
		PTH		-0.034	0.8190
		Magnesium	47	-0.071	0.6374
		Activin A	48	-0.019	0.9004
		Creatinine		+0.013	0.9314
		eGFR		-0.058	0.6942
		Pre-transplant dialysis duration		+0.114	0.4387
		Time since transplant		-0.047	0.7515
		Cumulative glucocorticoid dose	46	-0.069	0.6473
	IOP LE	Age	48	+0.003	0.9814
		BMI		-0.043	0.7698
		Corrected calcium		-0.055	0.7095
		Phosphate		+0.136	0.3582
		PTH		-0.003	0.9864
		Magnesium	47	-0.067	0.6547
		Activin A	48	+0.036	0.8084
		Creatinine		+0.067	0.6533
		eGFR		-0.139	0.3465
		Pre-transplant dialysis duration		+0.160	0.2777
		Time since transplant		+0.078	0.5993
		Cumulative glucocorticoid dose	46	-0.010	0.9486
	TBUT RE	Age	48	-0.121	0.4134
		BMI		+0.060	0.6858
		Corrected calcium		-0.377	0.0083
		Phosphate		+0.075	0.6135
		PTH		-0.143	0.3308
		Magnesium	47	-0.033	0.8256
		Activin A	48	-0.050	0.7340
		Creatinine		-0.115	0.4382
		eGFR		+0.028	0.8501
		Pre-transplant dialysis duration		+0.029	0.8453
		Time since transplant		+0.078	0.5972

Group	Parameter	Systemic/treatment-related variable	N	Spearman rho (ρ)	Nominal p-value
		Cumulative glucocorticoid dose	46	+0.061	0.6891
	TBUT LE	Age	48	-0.159	0.2792
		BMI		+0.068	0.6466
		Corrected calcium		-0.281	0.0530
		Phosphate		+0.077	0.6008
		PTH		-0.204	0.1639
		Magnesium	47	+0.008	0.9555
		Activin A	48	-0.008	0.9582
		Creatinine		-0.096	0.5145
		eGFR		+0.071	0.6318
		Pre-transplant dialysis duration		+0.054	0.7148
		Time since transplant		-0.018	0.9032
		Cumulative glucocorticoid dose	46	+0.063	0.6761
	Schirmer I RE	Age	48	-0.171	0.2449
		BMI		-0.072	0.6282
		Corrected calcium		-0.183	0.2135
		Phosphate		-0.063	0.6685
		PTH		-0.004	0.9771
		Magnesium	47	+0.101	0.4992
		Activin A	48	-0.045	0.7639
		Creatinine		-0.108	0.4659
		eGFR		+0.119	0.4191
		Pre-transplant dialysis duration		-0.050	0.7368
		Time since transplant		+0.128	0.3855
		Cumulative glucocorticoid dose	46	+0.068	0.6544
	Schirmer I LE	Age	48	-0.045	0.7601
		BMI		+0.029	0.8450
		Corrected calcium		-0.317	0.0279
		Phosphate		-0.075	0.6139
		PTH		-0.023	0.8772
		Magnesium	47	+0.080	0.5951
		Activin A	48	-0.001	0.9946
		Creatinine		-0.090	0.5428
		eGFR		+0.089	0.5452
		Pre-transplant dialysis duration		+0.079	0.5934
		Time since transplant		+0.067	0.6514
		Cumulative glucocorticoid dose	46	+0.191	0.2026

Note. Highlighted rows indicate nominal $p < 0.05$. These p-values are uncorrected for multiple testing and should be interpreted as exploratory.

Abbreviations: BCVA, best-corrected visual acuity; BMI, body mass index; DM, diabetes mellitus; eGFR, estimated glomerular filtration rate; HD, hemodialysis; IOP, intraocular pressure; KTR, kidney transplant recipients; LE, left eye; RE, right eye; TBUT, tear break-up time.
